# Influence of Rapid Heat Treatment on the Photocatalytic Activity and Stability of Barium Titanates Against a Broad Range of Pollutants

**DOI:** 10.3390/molecules29225350

**Published:** 2024-11-14

**Authors:** Mahsa Abedi, Haythem S. Basheer, Laura Lakatos, Ákos Kukovecz, Zoltán Kónya, Tamás Gyulavári, Zsolt Pap

**Affiliations:** 1 Department of Applied and Environmental Chemistry, University of Szeged, Rerrich Béla Sqr. 1, 6720 Szeged, Hungary; mahsa@chem.u-szeged.hu (M.A.); haythemsuliman@gmail.com (H.S.B.); lara.lakatos@gmail.com (L.L.); kakos@chem.u-szeged.hu (Á.K.); konya@chem.u-szeged.hu (Z.K.); 2 Nanostructured Materials and Bio-Nano-Interfaces Center, Interdisciplinary Research Institute on Bio-Nano-Sciences, Babes-Bolyai University, T. Laurian 42, 400271 Cluj-Napoca, Romania; 3 Centre for 3B, Babes-Bolyai University, Clinicilor 5–7, 400006 Cluj-Napoca, Romania

**Keywords:** photocatalysis, barium titanate, rapid calcination, water treatment, CO_2_ conversion

## Abstract

Barium titanate photocatalysts were synthesized via a sol–gel method involving a unique, cost-effective calcination technique that includes rapid heating and short exposure. The samples were characterized by X-ray diffractometry, scanning electron microscopy, diffuse reflectance spectroscopy, photoluminescence spectroscopy, infrared spectroscopy, and nitrogen adsorption–desorption measurements. The photooxidation activity and stability of the samples were evaluated by the degradation of phenol, oxalic acid, and chlorophenol. Their photoreduction activity was also investigated by the photocatalytic conversion of CO_2_ to CO. In both cases, UV irradiation was applied to activate the catalysts. As references, commercially available cubic and tetragonal barium titanates were used, with the addition of benchmark P25 TiO_2_ in some cases. Increasing the calcination temperature resulted in increased primary crystallite sizes, decreased specific surface areas, and slightly redshifted band gaps. On the one hand, the overall photooxidation activity of the samples for pollutant degradation was rather low, possibly due to their unfavorable valence band maximum position. On the other hand, our samples displayed significantly superior photoreduction activity, surpassing that of all the references, including P25 TiO_2_. The high photoactivity was mainly attributed to the specific surface areas that changed per the efficiency of the samples. Last, the cost comparison calculations showed that applying our calcination technique is 29.5% more cost-efficient than conventional calcination, and the same amount of energy is sufficient for preparing even a 1.4 times higher amount of barium titanite.

## 1. Introduction

Barium titanate (BTO), a semiconductor with a perovskite structure, is a material frequently utilized for its ferroelectric and piezoelectric properties [1]. Its advantageous characteristics, such as high oxygen vacancy concentrations, tunable size and morphology, spontaneous polarization, rapid migration of photogenerated charge carriers, and band bending [2,3], make it suitable for photocatalytic applications as well. BTO exhibits multiple crystal phases including cubic, tetragonal, orthorhombic, and rhombohedral, which exhibit ferroelectricity (except for the cubic phase) [4]. Ferroelectricity can influence photocatalytic activity and depends on various properties including size [5,6] and crystalline composition. Notably, the crystalline composition (phase transformations) is influenced by the way BTO is synthesized including parameters such as chelating agents [7] and calcination temperatures [6,8,9,10].

The sol–gel method is a flexible way to produce monodisperse, small crystals with high purity [11,12]. Furthermore, this method is inexpensive as it does not require special equipment [13]. Calcination is a critical step in the sol–gel process as it significantly influences the primary crystallite size, specific surface area, and crystallinity while modifying other parameters such as the morphology and band gap [14,15]. Pap et al. demonstrated that conventional calcination can be substituted with rapid calcination without significant alterations in the crystalline composition and other material properties [16]. This alternative method also yields comparable or slightly enhanced photoactivity, while also being advantageous from an economic perspective [14,17]. The phase composition of BTO undergoes significant changes as a function of the calcination temperature between 120 °C and 1457 °C. The cubic phase, characterized by Ti^4+^ and Ba^2+^ surrounded by an octahedral array of oxygen atoms, can be obtained at low calcination temperatures [13]. However, at high temperatures, BTO transitions to the tetragonal phase [7].

In this study, we investigate the photocatalytic degradation of organic pollutants (phenol, chlorophenol, and oxalic acid) and the conversion of CO_2_ using BTO photocatalysts prepared by applying a novel rapid calcination method. The effect of rapid calcination on the photocatalytic activity of pure BTO against such pollutants has not been investigated so far to our knowledge. We also aim to explore the relationship between the calcination temperature and the photocatalytic efficiency of BTO by examining its structural, morphological, and optical properties, including its stability against various functional groups.

## 2. Results and Discussion

### 2.1. Characterization of the Photocatalysts

#### 2.1.1. Crystal Phase Composition (XRD)

Initially, the synthesis of BTO photocatalysts was performed until obtaining a dried, amorphous gel. At this juncture, a thermogravimetric (TG) analysis was conducted to determine the temperature at which weight loss ceased (Figure S1). The analysis revealed significant weight losses associated with the removal of surface water, crystal water, and carbonaceous compounds derived from titanium(IV) butoxide. It was observed that the weight remained constant when ≥700 °C temperatures were applied. Based on these findings, the calcination of the samples was conducted at 700, 800, 900, and 1000 °C.

**Figure 1:**
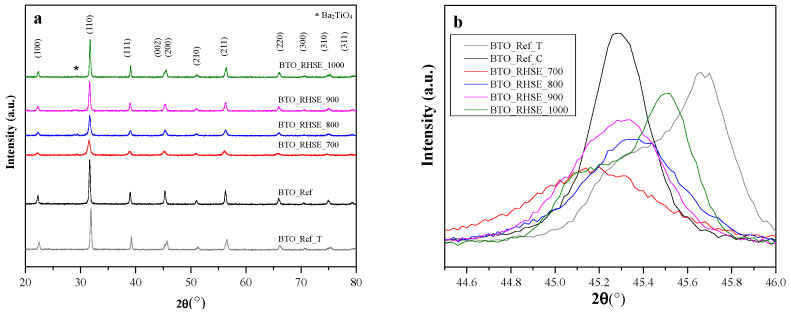
XRD patterns of BTO_Ref and BTO_RHSE samples calcined at different temperatures (**a**) and zoomed-in section between 44.5 and 46 2θ° demonstrating cubic and tetragonal structures (**b**).

Figure 1a illustrates the crystalline structure of the BTOs as determined by XRD analysis. It is important to highlight that the XRD patterns for cubic and tetragonal BTO are almost identical; the only slight difference is the splitting of the (200) peak in the latter case, reflecting the slight structural distortion [18]. The XRD patterns of BTO_RHSE_700, BTO_RHSE_800, and BTO_RHSE_900 align well with the standard cubic barium titanate phase (JCPDS No. 31-174) [19]: distinct diffraction peaks were observed at 22.3°, 31.7°, 38.9°, 45.3°, 50.9°, 56.1°, and 65.8°, which correspond to the Miller indices (100), (110), (111), (200), (210), (211), and (220), respectively [20]. To investigate a possible cubic–tetragonal phase transition, we recorded the XRD patterns in the 44.5–46.0° region at the highest possible resolution (Figure 1b), which confirmed that these three samples are indeed cubic. In BTO_RHSE_1000, the (200) diffraction peak split to a doublet at 45.5° ((200) plane) with a shoulder at 45.2°, the latter of which can be attributed to the (002) crystallographic plane of tetragonal BTO [21,22,23]. For comparison, the XRD patterns of the commercial BTO samples with cubic (BTO_Ref_C) and tetragonal (BTO_Ref_T) structures were also recorded. In BTO_Ref_C, only a single diffraction peak at 45.3° is visible. However, in BTO_Ref_T, the splitting of the (200) diffraction peak could be observed as expected, confirming its tetragonal structure [22], the same way as in BTO_RHSE_1000. The small reflections observed at 2θ = 28.9° and 29.5° are unique to the RHSE samples and could be assigned to the (211) and (031) planes of barium orthotitanate (Ba_2_TiO_4_), respectively [21,24].

Using the Scherrer equation [25], we calculated the average primary crystallite sizes. The results (Table 1) indicate a relationship between the calcination temperature and the crystallite size. As expected, the specific surface areas decreased with increasing calcination temperatures, which correspond with the observed growth in the primary crystallite sizes. The crystallinity degree of the BTOs increased with the increasing calcination temperatures, as expected. Within the BTO_RHSE samples, BTO_RHSE_1000 displayed the highest level of crystallinity, the largest primary crystallite size, and the lowest specific surface area, while the opposite was observed for BTO_RHSE_700. BTO_Ref_T showed very similar values compared to those of BTO_RHSE_1000, and, conversely, BTO_Ref_C exhibited a relatively large crystallite size while also maintaining a comparatively high surface area.

Stability measurements were also carried out by investigating the XRD patterns (Figure S2) of the samples after their exposure to the aqueous solutions of pollutants for 4 h. All the photocatalysts retained their crystalline composition, demonstrating their high structural stability under the tested conditions.

#### 2.1.2. Morphology (SEM)

**Figure 2:**
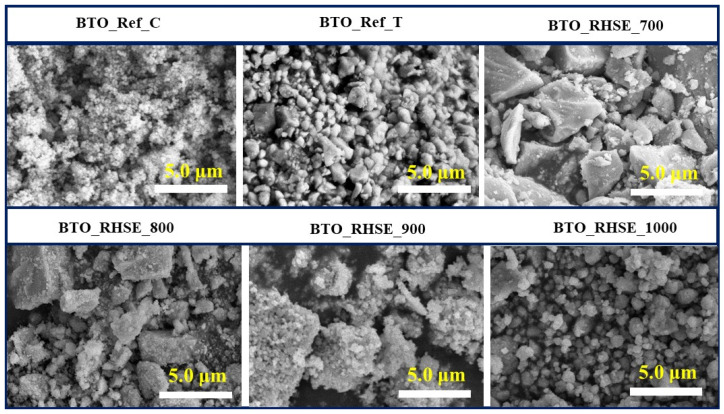
SEM micrographs of commercial BTO and BTO_RHSE photocatalysts calcined at various temperatures.

The morphology of samples can influence their photoactivity [26]; thus, SEM measurements were carried out and the results are shown in Figure 2. BTO_Ref_C is characterized by spherical particles with a low degree of aggregation. In contrast, BTO_RHSE_700 and BTO_RHSE_800 primarily consist of large, angular aggregates alongside smaller rounded particles. This trend reverses in BTO_RHSE_900 and BTO_RHSE_1000, where the large aggregates decrease, and smaller rounded particles become more prevalent, resembling the morphology observed for BTO_Ref_T.

As mentioned in the introduction, the tetragonal phase forms only at higher calcination temperatures [7,13], which was also the case in our BTO_RHSE_1000 sample. According to Stojanovic [27], and based on the study of Maison et al. [22], the dominant phase at relatively low calcination temperatures is cubic. These results are in good agreement with our findings. At lower temperatures, our samples initially exhibit large, angular aggregates. As the temperature increases, the extent of aggregation gradually decreases, resulting in the formation of smaller, rounded particles.

#### 2.1.3. Optical Properties

##### DRS

**Figure 3:**
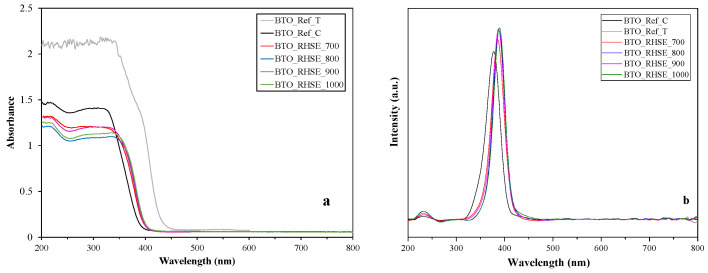
DR absorbance (**a**) and first-order derivative spectra (**b**) of the BTO_Ref and BTO_RHSE samples.

The absorbance (Figure 3a) and first-order derivative (Figure 3b) DR spectra of the samples were recorded to investigate the optical properties and calculate band gap values. The band gaps reported in the literature for BaTiO_3_ vary significantly, ranging between 3.0 and 3.6 eV [28,29]. The band gap values of our BTO_RHSE samples ranged from 3.18 to 3.21 eV (Table 1), which is close to the most frequently reported value (3.2 eV) [30]. A redshift could be observed as the band gaps expressed in wavelengths predominantly increased with increasing calcination temperature. This phenomenon has already been observed in our previous publications [14,31] and the literature [32] for TiO_2_. Since the difference in the band gap values of the samples is rather small, this will presumably not affect the photocatalytic activity significantly.

##### PL

**Figure 4:**
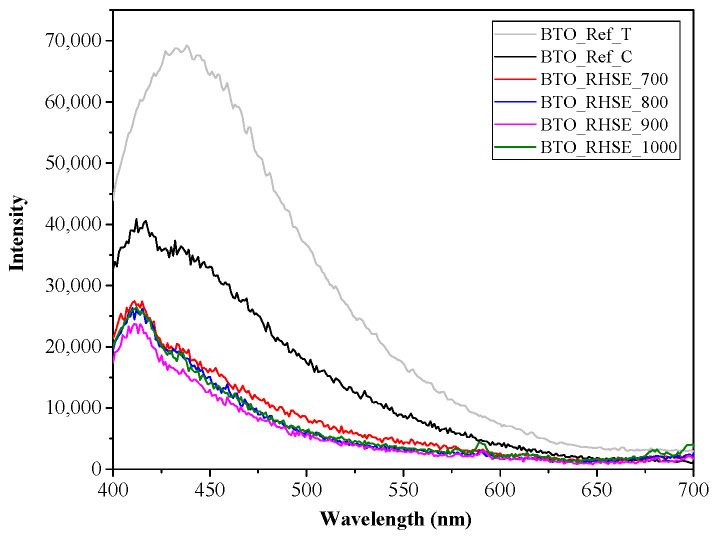
Photoluminescence spectrum for the BTO_Ref and BTO_RHSE samples.

Figure 4 shows the PL spectra of the BTO catalysts recorded using an excitation wavelength of 350 nm. The higher PL intensity of the reference samples indicates a lower degree of recombination compared to the homemade samples. All the samples exhibit a broad emission peak centered around ~412 nm, with an additional shoulder at ~433 nm. This wide band, which shows the highest intensity, is attributed to the direct recombination of electrons and holes [33,34]. The asymmetric shape of the spectrum, which is characterized by the small decrease in intensity over an even wider region at the lower energy side, suggests the presence of defect sites within the lattice [35]. Notably, there is no clear correlation between the emission intensity of the samples and their calcination temperature.

#### 2.1.4. Surface Properties

**Figure 5:**
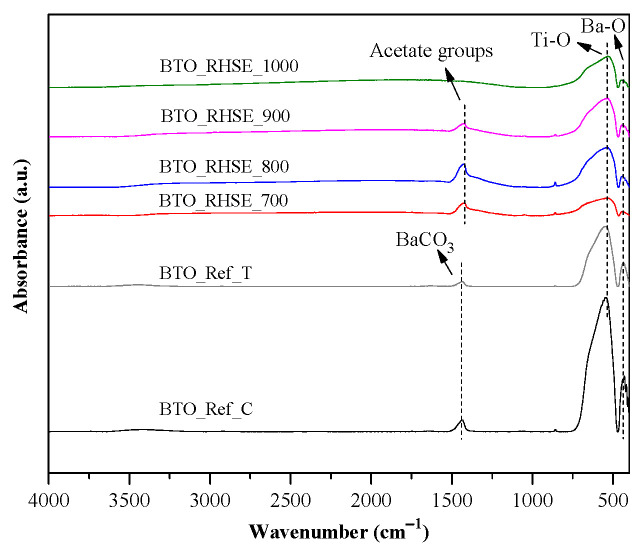
IR spectra of the BTO_Ref and BTO_RHSE samples.

IR spectroscopy measurements were conducted to investigate the surface properties of the samples, with the results presented in Figure 5. The absorption bands observed at wavenumbers below 800 cm^−1^ are attributed to the metal–oxygen (M–O) bonds [36,37,38]. Two types of metal oxide stretching vibrations were detected within the range of 430 to 770 cm^−1^. These absorption bands correspond to the presence of Ba–O and Ti–O bonds [39]. The stretching and bending vibrations of TiO_6_ were associated with the absorption band observed at approximately ~540 cm^−1^ [40,41]. The spectrum obtained for BTO_RHSE_1000 revealed a band at ~540 cm^−1^ with a shoulder at ~740 cm^−1^. This feature is consistent with the infrared spectra of tetragonal BTO powders as reported by several authors (although there is some variability depending on the particle size, shape, and aggregation) [19,42]. The small bands at 860 and 1043 cm^−1^ can be attributed to C–H bending vibrations [43,44]. Their appearance can be ascribed to carboxylate groups bonded to titanium, which disappear at 1000 °C due to decomposition [19]. The bands observed at 1421 cm^−1^ in the BTO_RHSE samples show acetate groups attached to barium [38,45], which originate from the precursor used for the synthesis (asymmetric and symmetric stretching vibrations of carboxylate groups also appear in this region). The absorption band around 1434 cm^−1^ in BTO_Ref_C and BTO_Ref_T is associated with BaCO_3_ [30,46].

### 2.2. Photocatalytic Activity

#### 2.2.1. Photocatalytic Oxidation

**Figure 6:**
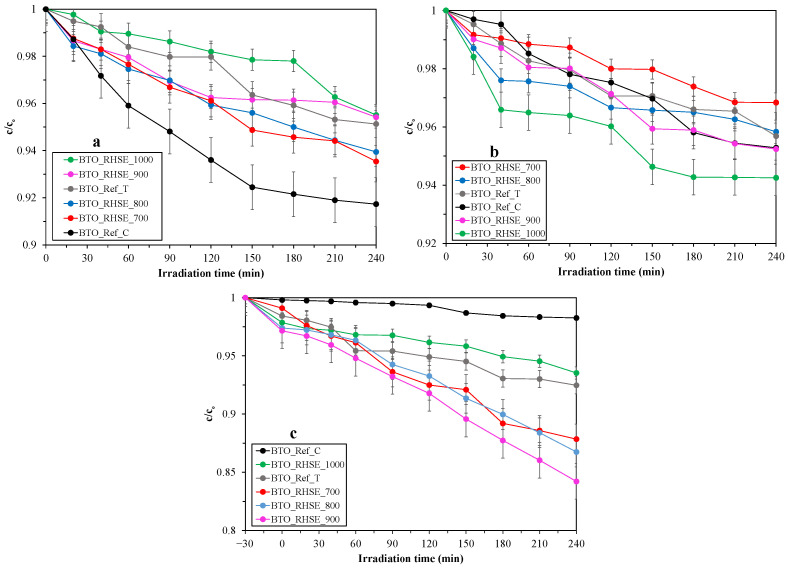
Degradation curves for (**a**) phenol, (**b**) chlorophenol, and (**c**) oxalic acid.

The photocatalytic performance of the BTO samples was evaluated through the degradation of three model pollutants: phenol (Figure 6a), chlorophenol (Figure 6b), and oxalic acid (Figure 6c). Before turning on the lamps, the photocatalysts were stirred in the dark to reach adsorption–desorption equilibrium. No adsorption was observed for phenol and chlorophenol, while the degree of adsorption was less than 3% for oxalic acid.

The photocatalytic oxidation activity of the samples is rather low in all cases (the conversions are lower than 10% for phenol and chlorophenol, and lower than 20% for oxalic acid). The low photooxidation activity makes the identification of the best sample and the investigation of individual differences uncertain, as most of the differences are comparable to the error of the HPLC technique. The overall higher photoactivity for oxalic acid degradation can be explained by the better adsorption property of oxalic acid. Comparatively, for phenol and chlorophenol, the lower photoactivities can be attributed to their low adsorption, which was also observed for other alkaline earth metal titanates and titanium dioxides in our previous studies [14,17,47,48,49,50]. The overall low conversions can be explained by the unfavorable position of the BTO valence band maximum for photocatalytic oxidation (Figure S3). This makes BTO less effective in generating some of the free radicals needed to oxidize organic pollutants. To support this statement, we carried out hydroxyl radical scavenger experiments using coumarin (the reaction of hydroxyl radicals with coumarin forms 7-hydroxycoumarin that can be detected via fluorescence measurements, and the amount of 7-hydroxycoumarin formed corresponds to the amount of hydroxyl radicals generated [51]). We found that our overall best sample, i.e., BTO_RHSE_700, forms ~10^−8^ M 7-hydroxycoumarin (Figure S4), proving that hydroxyl radical formation is severely unfavored with BaTiO_3_ (for comparison, for titanium dioxide, we measured ~two orders of magnitude higher 7-hydroxycoumarin concentrations [52]). It is also worth noting that Ba_2_TiO_4_ might also influence photoactivity. Information on the possible photocatalytic properties of Ba_2_TiO_4_ has not been reported so far; however, pure Ba_2_TiO_4_ has been investigated in luminescence [53] and photoelectrochemical [24] applications.

The BTO_RHSE_700 sample was selected to investigate its reusability in phenol degradation experiments over three cycles. Figure S5 shows that the differences in phenol conversions are within the error of the HPLC technique, highlighting the reusability of the sample.

#### 2.2.2. Photocatalytic Conversion of CO_2_

**Figure 7:**
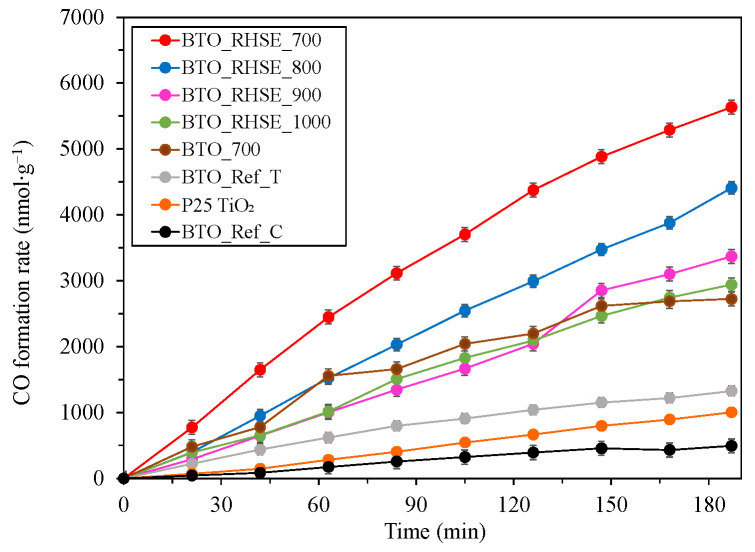
Photocatalytic conversion of CO_2_ using BTO_Ref, BTO_RHSE, and P25 TiO_2_ samples.

The photocatalytic activity of the samples was evaluated also by measuring the photocatalytic reduction of CO_2_ (Figure 7). As an additional reference, the well-known benchmark, P25 TiO_2_ was also considered. The selectivity for CO was consistently greater than 99.95% for all the samples. The conversion values are summarized in Table 1. Notably, BTO_Ref_C and BTO_Ref_T exhibited very low CO formation rates; lower than those of any of our BTO_RHSE samples. The same observation was made in the case of P25 TiO_2_. For our BTO_RHSE samples, significantly better CO_2_ conversion efficiencies were achieved: they increased consistently with the decreasing calcination temperatures used during the synthesis. This result is in good agreement with the specific surface areas that increased with decreasing calcination temperatures. A higher specific surface area provides more active sites for photocatalytic reactions, facilitating better interaction with the reactants, leading to increased CO formation [54]. The comparatively higher photoreduction activity of the samples compared to their photooxidation activity can be associated with their favorable conduction band minimum position [55,56,57] (Figure S3), enabling the following reaction:

CO_2_ + 2H^+^ + 2e^−^ → CO + H_2_O (1)

Our best-performing sample, BTO_RHSE_700, displayed over 5.5 times better photoreduction activity than the well-known commercial reference P25 TiO_2_. The overall best-performing sample, BTO_RHSE_700, was selected for investigating the reusability in the photoreduction experiments as well. Based on Figure S5, we found that the overall difference between the values was less than 0.7%, demonstrating the reusability of the samples.

### 2.3. Cost Efficiency Comparison of Conventional Calcination and the RHSE Method

In our earlier papers, we calculated how much more cost-effective our rapid calcination method is compared to conventional calcination in the case of strontium titanate [14] and calcium titanate [17] photocatalysts. The considerations, calculations, and equations are shown in detail in these publications and in the Supplementary Material. Accordingly, we carried out these calculations on barium titanate as well. For this purpose, we selected our overall best-performing sample (BTO_RHSE_700) and prepared its counterpart via conventional calcination (5 °C min^−1^ heating rate and 2 h exposure at 700 °C). This sample was named “BTO_700”. After carrying out the comparison, we found that the energy required to prepare BTO_700 is 1870 kW, while that required for synthesizing BTO_RHSE_700 is 1320 kW. We also compared BTO_700 and BTO_RHSE_700 in terms of the photoactivity (CO_2_ conversion efficiency) and found that the latter had twice as high a yield (Figure S6). Thus, applying our RHSE method is a suitable way to cut costs, while not only retaining but significantly increasing the photocatalytic activity.

Moreover, there are several other benefits of the RHSE method. BTO is a material with relatively high thermal conductivity among mixed oxides (i.e., 2.8 W m^−1^ K^−1^, which is higher than that of silicon: 2.3 W m^−1^ K^−1^). Micro- and nanostructures tend to have slightly lower thermal inertia compared to macroscopic materials [58]. This means that high thermal conductivity values and low thermal inertia lead to fast energy transfer within the material. By using the RHSE method, keeping the thermal properties of BTO in mind, it is safe to conclude that the xerogel receives the necessary heat required for the crystallization process (as confirmed by the XRD results). Moreover, the heat exchange is facilitated by convective heat transfer, which is characteristic of our system (the Reynolds number for the quartz tube in the furnace was calculated to be 11201, which is in the turbulent flow region) [59]. Having such a favorable heat transfer system, it can be ascertained that higher quantities of BTO can be prepared using the RSHE method. Specifically, the same amount of energy, i.e., 1320 kW, can be used for crystallizing even a 1.4 times higher amount of xerogel, thus optimizing the invested energy further.

## 3. Materials and Methods

### 3.1. Materials

Titanium(IV) butoxide (Ti(OC_4_H_9_)_4_; Sigma-Aldrich, Schnelldorf, Germany; reagent grade, ≥97%) and barium acetate (Ba(CH_3_COO)_2_; Alfa Aesar, Kandel, Germany; ACS grade, 99%) were used as BTO precursors. Ethanol absolute (VWR Chemicals, Debrecen, Hungary; Reag. Ph. Eur) was used as a solvent. Acetic acid (VWR Chemicals, Rosny-sous-Bois, France) was used as a chelating agent. Phenol (Spektrum 3D, Debrecen, Hungary; analytical grade, 100%), oxalic acid (Sigma-Aldrich, Germany; ≥99%), and chlorophenol (Sigma-Aldrich, Germany; reagent grade, ≥97%) were used for photocatalytic activity measurements. A gas mixture of CO_2_:H_2_ = 1:2 was used to evaluate the photoreduction activity of the samples. Commercial BTO (Sigma-Aldrich, Germany; ≥99% and Thermo Fisher Scientific, Waltham, MA, USA; 99.7%), and Aeroxide P25 TiO_2_ (Acros Organics, Waltham, MA, USA) were used as references.

### 3.2. Synthesis of the Photocatalysts

BTO powders were produced through a sol–gel method based on the publication of Li et al. [6]. The synthesis process comprised the following steps: an organic medium (A) consisting of 0.1 mol Ti(OC_4_H_9_)_4_, 0.6 mol absolute ethanol, and 0.3 mol glacial acetic acid was prepared through stirring at room temperature. Following this, an aqueous solution (B) of Ba(CH_3_COO)_2_ was formed by dissolving 0.1 mol Ba(CH_3_COO)_2_ in 90 mL of 36% acetic acid. Then, solution (B) was vigorously mixed with solution (A), obtaining a stoichiometric sol (Ba:Ti molar ratio = 1:1), followed by vigorous stirring for 1 h. Finally, the mixture was stirred for an additional 4 h at a lower stirring rate, resulting in the formation of a transparent gel at room temperature.

After the sol–gel process, the obtained amorphous powder was placed inside a ceramic evaporating dish and heated to 100 °C for 24 h in a Memmert UNB500 (Schwabach, Germany) drying oven. A unique calcination technique referred to as “rapid heating–short exposure” (RHSE) was used based on the publication of Pap et al. [16]. Three consecutive heating steps with a high heating rate (60, 20, and 10 °C min^−1^) were taken for this purpose. Reducing the heating rate as the temperature became closer to the desired temperatures (650, 700, 800, 900, and 1000 °C) prevented overheating. The samples were kept at the desired temperature for 5 min in a tube furnace (Thermolyne 21100, Waltham, MA, USA; overall length 38 cm, quartz tube length 64 cm, tube interior diameter 5.5 cm, quartz tube diameter 4 cm). Air was supplied continuously (0.5 L∙min^−1^) during the calcination process. The samples were ground in an agate mortar following the calcination process.

The samples we synthesized were named “BTO_RHSE_X”, where “X” corresponds to the temperature applied during calcination, while the “BTO_Ref_C” and “BTO_Ref_T” names were used for the cubic (Sigma-Aldrich) and tetragonal (Thermo Fisher Scientific) references, respectively.

### 3.3. Characterization Methods and Instrumentation

The following parameters were set using a Rigaku Miniflex II diffractometer (Rigaku, Neu-Isenburg, Germany) for X-ray diffraction (XRD) measurements: λ_Cu Kα_ = 0.15406 nm, 30 mA, 40 kV, 20–80 (2θ°) region. The Scherrer equation was used for calculating the average crystallite sizes of the catalysts. Using a Hitachi S-4700 Type II microscope (Hitachi, Tokyo, Japan) with an acceleration voltage of 10 kV, we captured scanning electron microscopy (SEM) images to assess the morphology of the material. Nitrogen adsorption–desorption tests via a NOVA 3000e device (Boynton Beach, FL, USA) and the Brunauer–Emmett–Teller (BET) method were carried out to determine specific surface areas [60]. Diffuse reflectance (DR) spectra of the materials were recorded using a Jasco-V650 Shimadzu UV-3600 Plus UV–vis–NIR (Shimadzu Corporation, Kyoto, Japan) spectrophotometer equipped with an ILV-724-type integration sphere to investigate their optical characteristics. The first-order derivative spectra were used as the basis for the calculation of the band gap energies of the samples. The photoluminescence (PL) emission spectra of the samples at room temperature were measured using a Horiba Jobin Yvon Fluoromax-4 spectrofluorometer (Horiba, Kyoto, Japan) with a 350 nm excitation wavelength and a 350 nm cut-off filter. To determine the minimum temperature required for calcination, a thermal gravimetric (TG) investigation of the xerogel was carried out using TGA Q500 (TA Instruments, New Castle, DE, USA) equipment. A heating rate of 10 °C min^−1^ was utilized within the 0–900 °C range. Using a Bruker Vertex 70 FTIR instrument (Bruker, Billerica, MA, USA), we obtained the Fourier transform infrared (FTIR) spectra of the samples at a resolution of 4 cm^−1^ in the wavenumber range of 400 to 4000 cm^−1^. For analysis, the samples were combined with KBr and compressed into thin pellets.

### 3.4. Determination of Photocatalytic Activities

Phenol (c_0, phenol_ = 0.1 mM), chlorophenol (c_0, chlorophenol_ = 0.1 mM), and oxalic acid (c_0, oxalic acid_ = 5 mM) were photocatalytically degraded to measure the photoactivity of the samples. In a controlled setting, six UV fluorescent tubes (Novelite, Bandung, Indonesia; UV-A, 6 W) were utilized for irradiation (λ_max_ = 365 nm). The suspensions of the photocatalysts were put within a double-walled glass vessel. Throughout the 4 h of the experiment, the temperature was maintained at 25 °C, and air was introduced into the reactor to maintain the dissolved oxygen content. High-performance liquid chromatography (HPLC) was used to monitor changes in the molar concentration of the pollutants. A Merck-Hitachi L-7100 (Hitachi, Rahway, NJ, USA) low-pressure gradient pump and a Merck-Hitachi L-4250 UV-vis detector comprised the chromatograph. The stability of the samples against compounds with different functional groups (phenolic hydroxyl, carboxylic, halide, alcoholic hydroxyl, and aldehyde groups) was also investigated, which is shown in the Supplementary Material.

The photocatalytic CO_2_ reduction experiments were conducted using a flow microreactor featuring two concentric glass (quartz) cylinders: an inner cylinder (height = 25 cm, diameter = 6.4 cm) and an outer cylinder (height = 25 cm, diameter = 10.2 cm). IV illumination was provided by an 11 W mercury vapor lamp (Heraeus Noblelight TQ 718, Hanau, Germany; λ_max_ = 254 nm). The light intensities on the photocatalytic surface were measured using a Quantum meter (Apogee, Santa Monica, CA, USA, Model MQ-200), resulting in 40 µmol m^−2^ s^−1^. To prepare the catalyst, we ultrasonically dispersed 250 mg of the sample in 10 mL of absolute ethanol, and this mixture was then immobilized onto the outer surface of the inner quartz cylinder. The catalyst film was pretreated at 250 °C with a heating rod while introducing various gases sequentially: Ar for 20 min, O_2_ for 30 min, Ar again for 10 min, and H_2_ for 60 min. The reaction was carried out using a CO_2_ gas mixture at a 1:2 ratio, controlled by a mass flow controller (Aalborg). This gas mixture was recirculated between the reactor and a gas chromatograph (GC) using a pump. During the experiments, the temperature was kept constant by recirculating cooling water. The identification and separation of the reactants and products were performed using an HP 5890 Series II GC (Palo Alto, CA, USA) equipped with a packed Porapak QS column (SKC, Blandford Forum, UK; diameter = 0.635 cm, length = 2 m). Products were detected using a thermal conductivity detector and a flame ionization detector.

## 4. Conclusions

The rapid calcination of barium titanate offers a promising approach to enhance its photoreduction activity while reducing the energy required for its synthesis. This method led to changes in the primary crystallite sizes (16.8 to 33.4 nm), accompanied by a decrease in specific surface areas (14.9 to 2.9 m^2^ g^−1^), and a slight redshift in the band gaps (~0.02 eV) as a function of temperature (700 to 1000 °C). Despite these changes, the material maintains its structural stability when exposed to various functional groups such as phenolic/alcoholic –OH, –Cl, –COOH, and –CHO groups, demonstrating its robustness in practical applications. While the photooxidation activity of barium titanate is relatively low (<20% conversions in all cases), presumably due to its unfavorable valence band maxi-mum, the material excels in photoreduction processes. Specifically, it shows superior activity in the photoreduction of CO_2_ to CO (as high as 179 nmol g^−1^ min^−1^), outperforming the commercial barium titanates (19–38 nmol g^−1^ min^−1^) and even the well-known photo-catalyst P25 TiO_2_ (33 nmol g^−1^ min^−1^). The increasing photoreduction performance is in good agreement with the increasing specific surface areas. Applying the RHSE method, energy-related costs can be cut significantly, while obtaining the same or even higher quality material. For the success of this calcination approach, convective heat transfer and acceptable thermal conductivity values are needed. Furthermore, rapid calcination presents not only an energy-efficient but also a performance-enhancing alternative for synthesizing barium titanate photocatalysts for CO_2_ reduction. By decreasing the required energy input, rapid calcination presents a more cost-effective and sustainable alternative to traditional heat treatments.

## Figures and Tables

**Table 1:** Average primary crystallite sizes, band gaps, specific surface areas, and CO_2_ conversion efficiencies of the investigated samples.

Sample Name	Crystallite Size (nm)	Specific Surface Area (m²·g^−1^)	Band Gap1st_,der_ (eV)	Band Gap_KM_ (eV)	CO Yield (nmol·g^−1^·min^−1^)
BTO_RHSE_700	16.8	14.9	3.20	3.03	179
BTO_RHSE_800	23.7	12.5	3.19	3.08	139
BTO_RHSE_900	28.5	4.2	3.19	3.06	110
BTO_RHSE_1000	33.4	2.9	3.18	3.00	94
BTO_Ref_C	31.8	10.6	3.28	3.16	19
BTO_Ref_T	29.3	2.7	3.24	3.13	38
P25 TiO_2_	25.4	49.6	3.11	3.17	33

## Data Availability

Data are contained within the article or Supplementary Material.

## Supplementary Materials

The following supporting information can be downloaded at https://www.mdpi.com/article/10.3390/molecules29225350/s1: Figure S1: Thermal gravimetric analysis (TGA); Figure S2: XRD patterns of BTO_RHSE samples and BTO_Ref_C after (a) phenol, (b) chlorophenol, and (c) oxalic acid degradations; Figure S3: Energy level diagram of anatase TiO_2_, CaTiO_3_, SrTiO_3_, and BaTiO_3_. CB represents the conduction band, and VB is the valence band; Figure S4: 7-hydroxycoumarin formation on BTO_RHSE_700; Figure S5: Reusability of BTO_RHSE_700 investigated for phenol degradation (left) and CO_2_ conversion (right) over three cycles; Figure S6: Comparison of CO_2_ conversion activity between BTO_700 and BTO_RHSE_700.
