# Supplementary material for: Influence of Rapid Heat Treatment on the Photocatalytic Activity and Stability of Barium Titanates Against a Broad Range of Pollutants
Source: Molecules. 2024 Nov 14;29(22):5350. doi: 10.3390/molecules29225350 (PMC11596716; doi:10.3390/molecules29225350)

molecules

MDPI

Supplementary material

Influence of rapid heat treatment on the photocatalytic activity and stability of barium titanates against a broad range of pollutants

Mahsa Abedi[1], Haythem S. Basheer[1], Laura Lakatos[1], Ákos Kukovecz[1], Zoltán Kónya[1], Tamás Gyulavári[1,*], Zsolt Pap[1,2,3,*]

[1] Department of Applied and Environmental Chemistry, University of Szeged, Rerrich Béla Sqr. 1, 6720 Szeged, Hungary
[2] Nanostructured Materials and Bio-Nano-Interfaces Center, Interdisciplinary Research Institute on Bio-Nano-Sciences, Babes-Bolyai University, T. Laurian 42, 400271 Cluj-Napoca, Romania
[3] Centre for 3B, Babes-Bolyai University, Clinicilor 5–7, 400006 Cluj-Napoca, Romania
* Correspondence: gyulavarit@chem.u-szeged.hu (T.G.); pzsolt@chem.u-szeged.hu (Z.P.); Tel.: +36-62-544-626 (T.G.); +36-62-544-316 (Z.P.)

Figure S1

Thermal gravimetric analysis (TGA) of the xerogel obtained by the end of the synthesis.

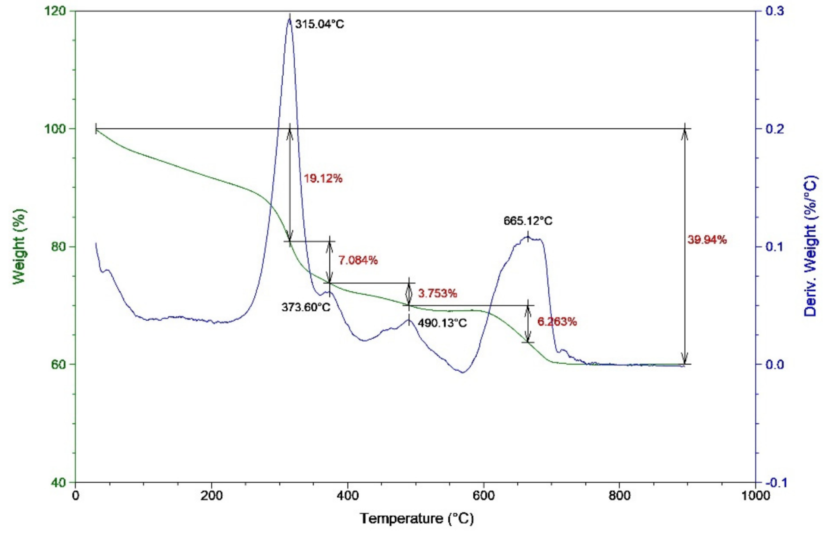

Figure S2

XRD patterns of BTO_RHSE samples and BTO_Ref_C after (a) phenol, (b) chlorophenol, and (c) oxalic acid degradations.

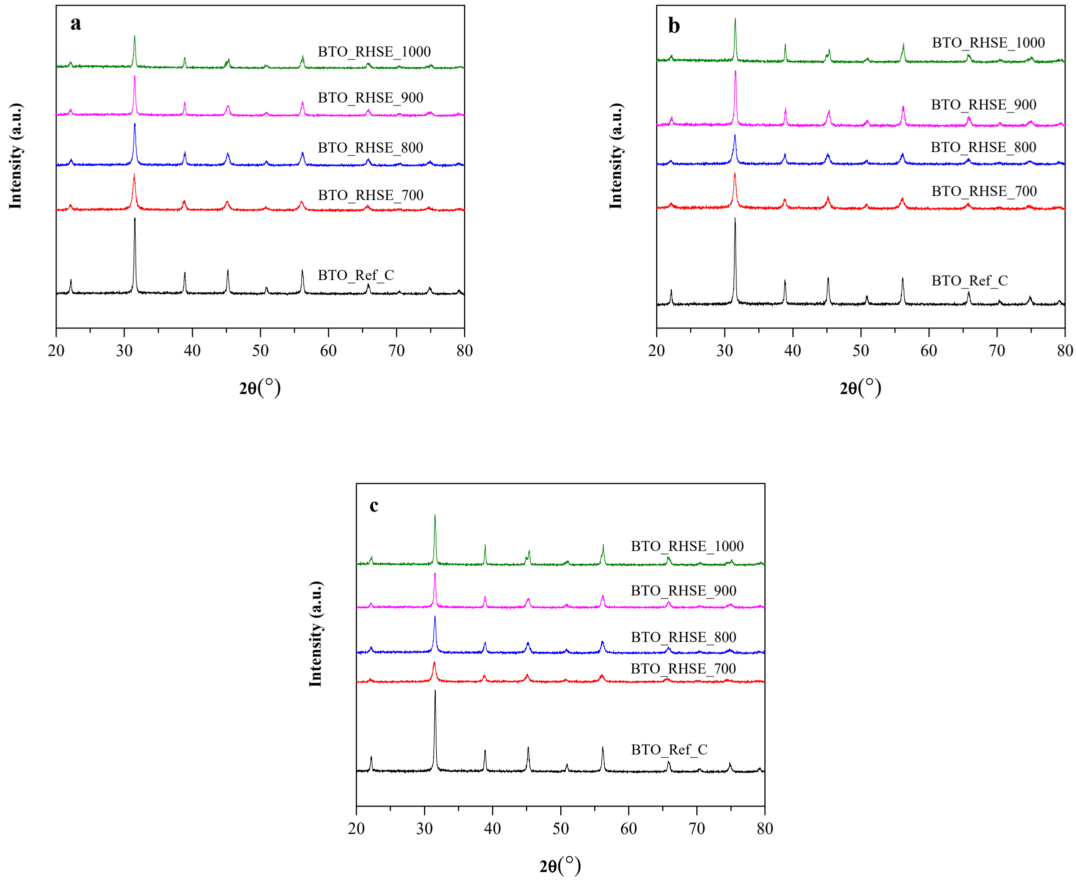

In addition to the mentioned model pollutants, alcoholic hydroxyl groups (propanol; VWR Chemicals, Hungary; Reag. Ph. Eur.), and aldehyde groups (glucose; VWR Chemicals, Hungary; Reag. Ph. Eur.) were also considered to evaluate the stability of the homemade catalyst. The stability test was conducted in the same manner as for the other pollutants, following 4 hours of degradation under UV light. The XRD patterns of BTO_RHSE_700 and BTO_Ref_C after (d) glucose and (e) propanol degradations are presented below. In neither case did the crystalline composition change following exposure to these materials.

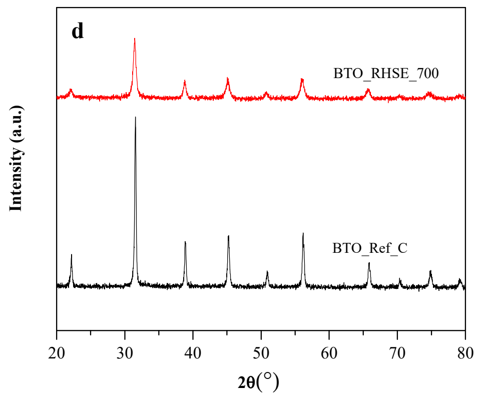

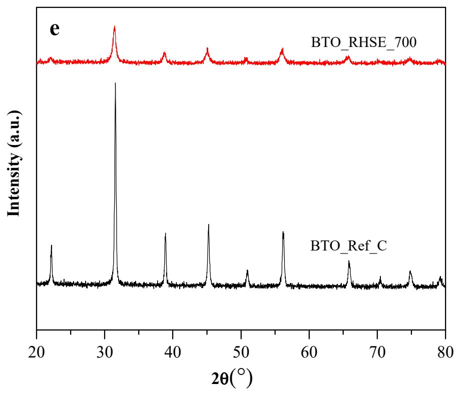

Figure S3

Energy level diagram of TiO_2, $CaTiO_3$, $SrTiO_3$, and $BaTiO_3$.

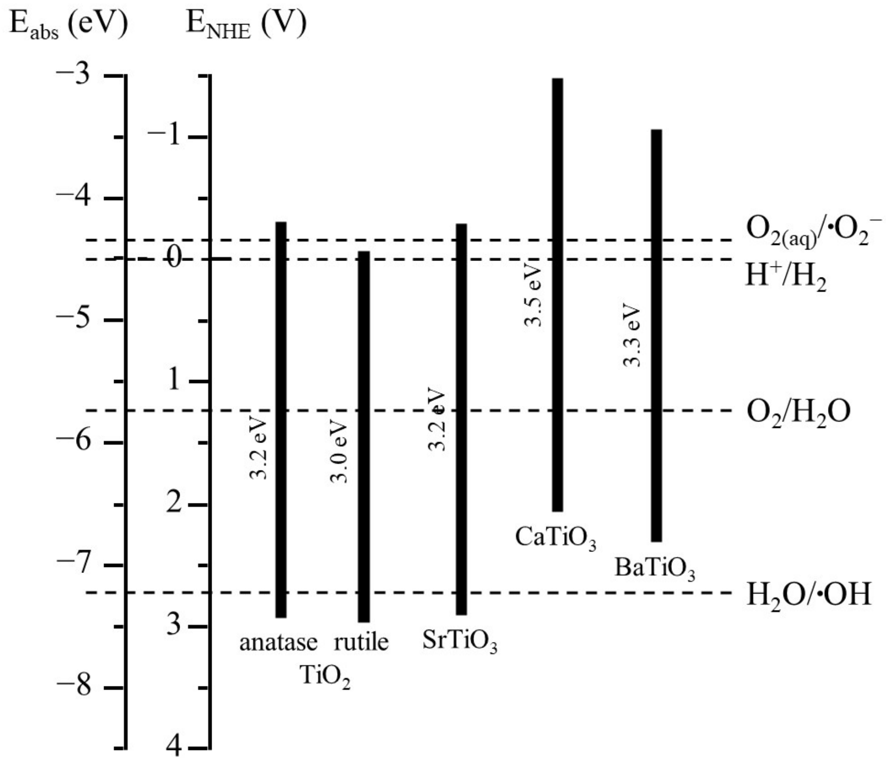

Figure S4

7-hydroxycoumarin formation on BTO_RHSE_700.

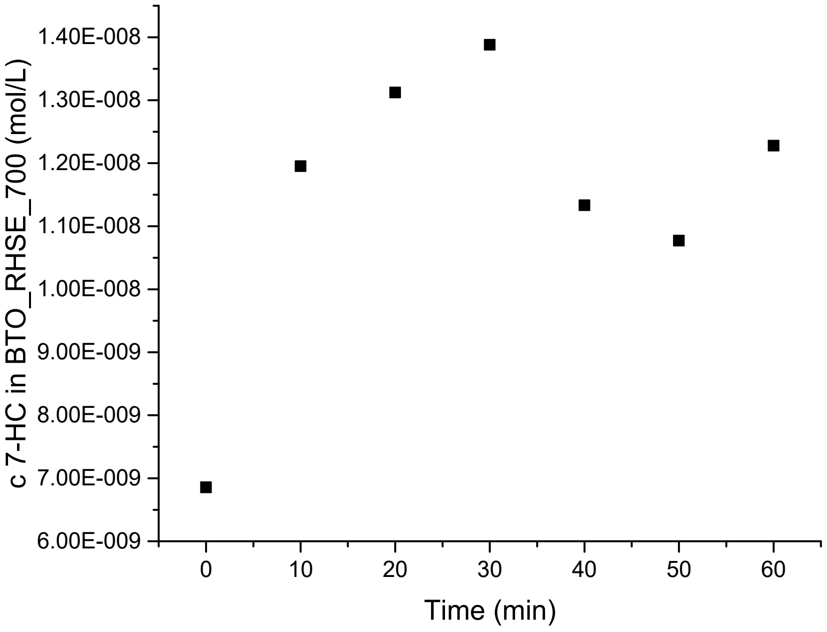

Figure S5

Reusability of BTO_RHSE_700 investigated for phenol degradation (left) and CO_2 conversion (right) over three cycles.

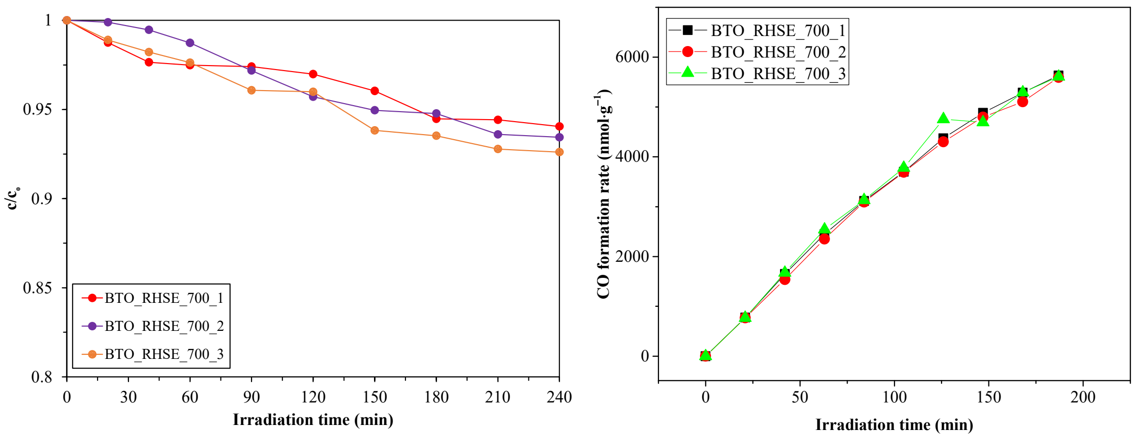

Energy calculations for heat transfer and power consumption

The calculations were based on the following considerations:

- The sample had a surface area of 1 cm^2 and a depth of 1 mm.
- Heat transfer occurred via convection, with natural convection being negligible (based on Nusselt and Reynolds numbers).
- Heat transfer was limited to the sample surface.
- Both BTO_RHSE_700 and BTO_700, were prepared using the same starting materials, so differences in chemical properties (enthalpy, entropy, etc.) were disregarded.

The energy required for synthesis (P) was calculated using the following equations, where T is the temperature, υ is the vectorial sum of hydrodynamic parameters, and j is the sum of intrinsic properties (assumed to be zero):

$$P = \nabla(T, v, j)$$

$$\nabla T = \frac{\partial T}{\partial x} + \frac{\partial T}{\partial y} + \frac{\partial T}{\partial z}$$

With one-dimensional heat transfer and equal air flux, the power consumption depends only on the temperature change and the equilibrium between the furnace temperature (700 °C) and airflow. By integrating the heating curve (temperature vs time), the electrical power for heating was calculated, while the heat loss equation via convection provided the energy required to maintain the temperature.

Figure S6

Comparison of CO_2 conversion activity between BTO_700 and BTO_RHSE_700.

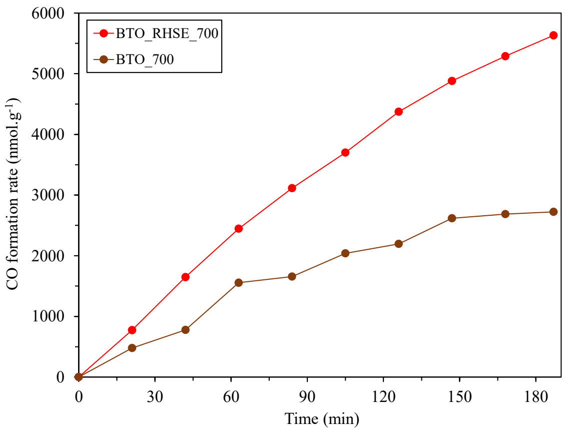

Supplement: Supplementary file 1 [file molecules-29-05350-s001.zip › molecules-3274410-supplementary.pdf]
